# Impact of sequencing depth and technology on de novo RNA-Seq assembly

**DOI:** 10.1186/s12864-019-5965-x

**Published:** 2019-07-23

**Authors:** Jordan Patterson, Eric J. Carpenter, Zhenzhen Zhu, Dan An, Xinming Liang, Chunyu Geng, Radoje Drmanac, Gane Ka-Shu Wong

**Affiliations:** 1 Department of Medicine, University of Alberta, Edmonton, AB T6G 2E1 Canada; 2 Department of Biological Sciences, University of Alberta, Edmonton, AB T6G 2E9 Canada; 3 MGI, BGI-Shenzhen, Shenzhen, 518083 China

**Keywords:** Rna-seq assembly, Sequencing depth, Sequencing technology

## Abstract

**Background:**

RNA-Seq data is inherently nonuniform for different transcripts because of differences in gene expression. This makes it challenging to decide how much data should be generated from each sample. How much should one spend to recover the less expressed transcripts? The sequencing technology used is another consideration, as there are inevitably always biases against certain sequences. To investigate these effects, we first looked at high-depth libraries from a set of well-annotated organisms to ascertain the impact of sequencing depth on de novo assembly. We then looked at libraries sequenced from the Universal Human Reference RNA (UHRR) to compare the performance of Illumina HiSeq and MGI DNBseq™ technologies.

**Results:**

On the issue of sequencing depth, the amount of exomic sequence assembled plateaued using data sets of approximately 2 to 8 Gbp. However, the amount of genomic sequence assembled did not plateau for many of the analyzed organisms. Most of the unannotated genomic sequences are single-exon transcripts whose biological significance will be questionable for some users. On the issue of sequencing technology, both of the analyzed platforms recovered a similar number of full-length transcripts. The missing “gap” regions in the HiSeq assemblies were often attributed to higher GC contents, but this may be an artefact of library preparation and not of sequencing technology.

**Conclusions:**

Increasing sequencing depth beyond modest data sets of less than 10 Gbp recovers a plethora of single-exon transcripts undocumented in genome annotations. DNBseq™ is a viable alternative to HiSeq for de novo RNA-Seq assembly.

## Background

RNA-Seq is a widely used next-generation sequencing (NGS) methodology for transcriptome profiling [1], both to identify novel transcript sequences and for differential expression studies. Much has been written about this methodology and it is not our intention to rehash the many excellent articles that can be found in the literature [2, 3]. We focus instead on how continuing improvements in NGS technologies have brought new perspectives to two fundamental questions that many scientists ask before they initiate a RNA-Seq experiment. First, with the decreasing costs of generating these data, one can now sequence a given library many times deeper than before. Motivated by ongoing projects on the sequencing of phylodiverse species with no reference genomes, e.g. 1KP for plants [4] and 1KITE for insects [5], we wanted to see how many novel transcript sequences can be recovered by de novo assemblies of RNA-Seq data if a project is willing to spend more money. This is not an issue that will soon be mooted by the ever lower costs of sequencing complete genomes, because genome size variations (e.g. 2,342-fold for land plants [6]), polyploidy, and the outbred nature of many samples collected in the wild make genome assembly a continuing challenge.

**Fig. 1:**
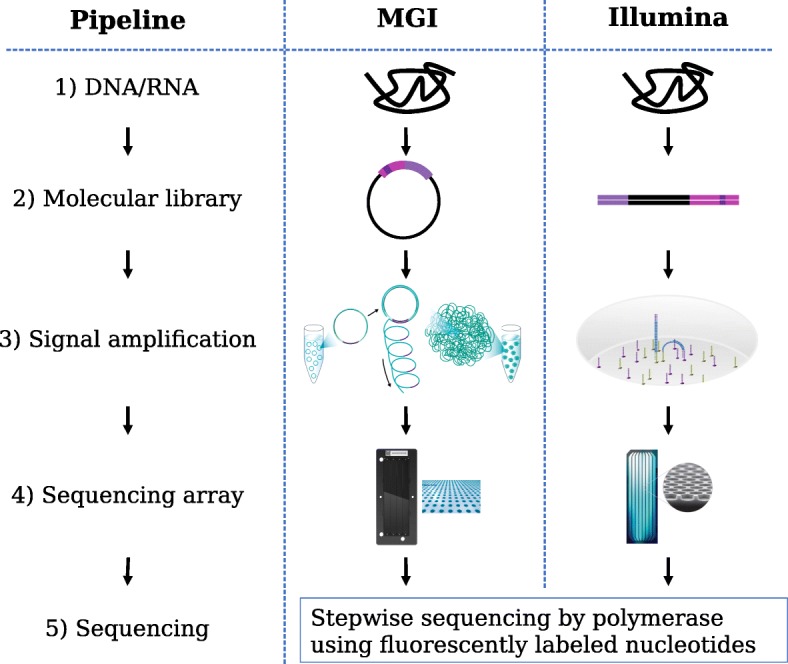
Technical comparison of DNBSeq™ and Illumina platforms

Choice among sequencing platforms is the second issue to be addressed. We will explore how DNBSeq™, a recent platform from MGI (a subsidiary of BGI Group), may serve as an alternative to the market-leading platform from Illumina. It uses DNA nanoballs (DNB) and combinatorial probe-anchor synthesis (cPAS) [7], building on technology from Complete Genomics. Both platforms provide short but high-quality reads, in contrast to the long but low-quality reads offered by Pacific Biosciences and Oxford Nanopore. There are several technical differences in the two sequencing pipelines, which are illustrated in Fig. 1.

In both platforms, DNA molecules (1) are fragmented. Adapters are ligated to these fragments and are processed to produce libraries (2) containing single-strand DNA circles with an adapter in the middle for DNBSeq™ and linear double stranded DNA with adapters at each end for Illumina. These DNA sequences are then replicated to produce an amplified signal for sequencing (3), using rolling circle replication for DNBSeq™ (producing DNBs) and bridge PCR amplification for Illumina (producing clusters). Rolling circle replication is a linear amplification where each replicate is generated from the original fragment and does not produce detectable clonal amplification errors or molecular switching of sample barcodes [8], produces a small percentage of spot duplicates, and has reduced coverage bias, resulting in better coverage of some GC-rich regions. The exponential amplification performed on Illumina libraries is known to have issues with molecular barcode switching [9] and GC-rich sequence coverage [10–12]. DNBSeq™ and newer Illumina instruments use patterned flow cells (4) for higher nanoball / cluster density. Both platforms use stepwise sequencing by polymerase on ssDNA template with fluorescently labeled nucleotides (5). The small size of the DNBs (~ 200 nm) relative to PCR clusters results in smaller and more concentrated DNA spot fluorescence, giving DNBSeq™ a higher signal-to-noise ratio, higher spot densities, and faster sequencing times.

DNBseq™ has also been investigated for use in analyzing small non-coding RNAs [7], palaeogenomic sequencing [13], metagenomic sequencing [14], germline and somatic variant identification in whole genomes [15], and transcriptome analysis in plants [16].

## Analyses

### Increased sequencing depth enriches for single-exon transcripts not reported in genome annotations

Sequencing depth is an important consideration for RNA-Seq because of the tradeoff between the cost of the experiment and the completeness of the resultant data. Transcript abundance follows an exponential distribution, and greater sequencing depths are required to recover less abundant transcripts. As more sequencing is done to assemble less abundant transcripts, a greater proportion of the additional reads will come from transcripts that already have sufficient depth to assemble. Hence there are diminishing returns as sequencing depth increases, and intuitively one might expect the number of recovered transcripts to asymptote. What happens in practice is surprising, as we will show for our representative species.

### Datasets

RNA-seq datasets are usually a few Gbp in size, but in the public databases there are some unusually large datasets with sizes in the many 10’s of Gbp. Importantly for our purposes, these were sequenced from a single library, not by pooling sequences from multiple libraries. We could therefore sub-sample these datasets to simulate the consequences of doing RNA-seq at varying sequencing depths. To benchmark the resultant assemblies, we used species with longstanding (decade old) reference genome annotations, i.e. *Homo sapiens*, *Mus musculus*, *Drosophila melanogaster*, *Caenorhabditis elegans, Oryza sativa*, *Arabidopsis thaliana*. Tables 1 and 2 show the datasets used and their related genomes, respectively. The data was downloaded from the National Center for Biotechnology Information’s (NCBI) Sequence Read Archive (SRA).

**Table 1:** Datasets used to study effect of sequencing depth

SRA Run ID	Species	Tissue Description	Platform	# of Spots	# of Bases	Date Published	End Length
SRR1047863	*Homo sapiens*	post-mortem brain (dorsolateral prefrontal cortex / Brodmann area 46)	Illumina HiSeq 1000	258 733 827	52 264 233 054	2014-01-08	101 bp
SRR980471	Homo sapiens	CD19+ primary cells, hispanic male age 37	Illumina HiSeq 2000	263 034 155	39 981 191 560	2013-09-12	76 bp
SRR1732347	*Mus musculus*	male brain age 8 weeks, strain C57BL/6 J	Illumina HiSeq 2000	354 274 087	71 563 365 574	2014-12-23	101 bp
SRR1509508	*Drosophila melanogaster*	adult; strain: y; cn bw sp	Illumina HiSeq 2000	140 645 540	28 410 399 080	2014-07-09	101 bp
SRR1523365	*Caenorhabditis elegans*	at least 100 adult worms	Illumina HiSeq 2000	228 557 939	45 711 587 800	2014-07-31	100 bp
SRR1178906	*Oryza sativa*	panicle from *O. sativa* japonica (Nipponbare cultivar)	Illumina HiSeq 2000	207 489 217	41 497 843 400	2014-03-03	100 bp
DRR018424	*Arabidopsis thaliana*	4 day old seedlings	Illumina HiSeq 2000	192 531 285	38 891 319 570	2014-11-27	101 bp
SRR1061361	Arabidopsis thaliana	leaves – when first flower open	Illumina HiSeq 2000	202 019 334	40 807 905 468	2015-07-22	101 bp

All datasets were sourced from the NCBI and DDJB Sequence Read Archives

**Table 2:** Reference genome and annotation (GFF) sources

Species	RefSeq Assembly and Annotation	Genome (bp)	Total Gaps (bp)	Exome (bp)
Homo sapiens	GCF_000001405.29_GRCh38.p3	3 226 010 022	161 368 151	120 562 222
Mus musculus	GCF_000001635.24_GRCm38.p4	2 803 568 840	79 356 756	114 986 282
Drosophila melanogaster	GCF_000001215.4_Release_6_plus_ISO1_MT	143 726 002	1 152 978	35 879 647
Caenorhabditis elegans	GCF_000002985.6_WBcel235	100 286 401	0	26 801 799
Oryza sativa	GCF_000005425.2_Build_4.0	382 778 125	10 060 004	49 757 833
Arabidopsis thaliana	GCF_000001735.3_TAIR10	119 667 750	185 644	57 812 822

All genome sequence and GFF reference files were obtained from the NCBI Assembly database

#### Quality control

All libraries were preprocessed with Trim Galore! to remove adapter/primer sequences. Only reads aligning to the host genome using TopHat2 [17] were kept, to prevent confounding effects from library contaminations. Random sampling of each library was done at sequencing depths of 1, 2, 3, 4, 5, 6, 8, 10, 12, 14, and 16 Gbp.

#### Assembly results

**Fig. 2:**
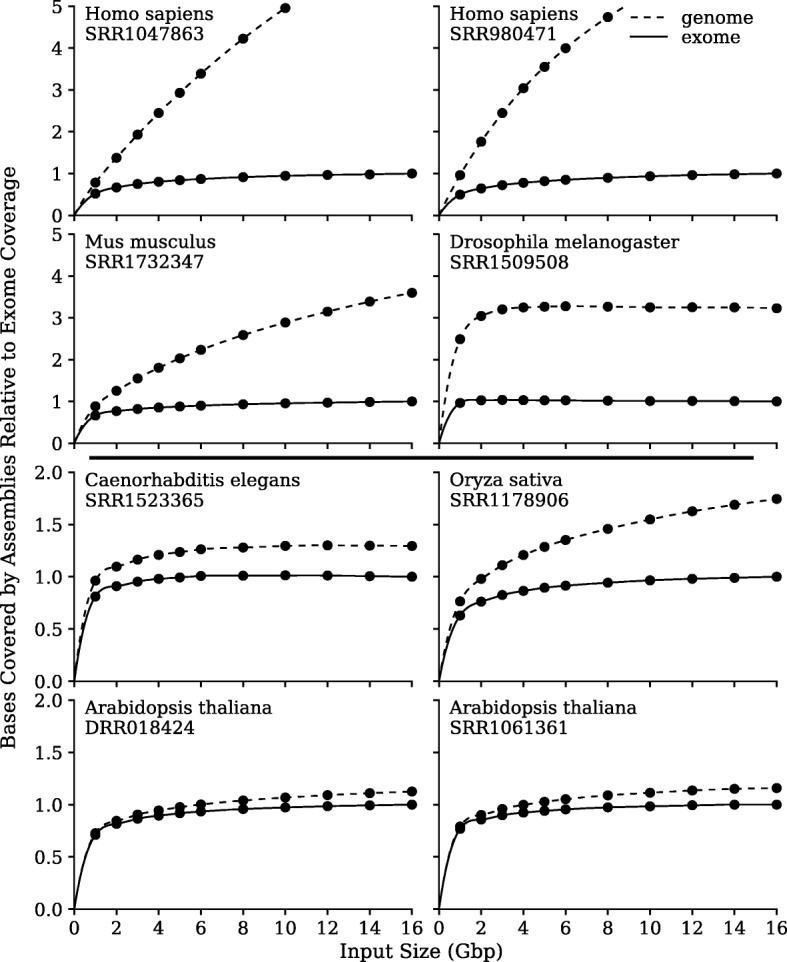
Effect of sequencing depth on transcriptome recovery. We count the total number of unique bases in the alignments, based either on the genome or the exome. The vertical scale normalizes the exome size of the 16 Gbp assemblies to unity

Each sub-sampled dataset was de novo assembled by SOAPdenovo-Trans and gap-filled using GapCloser [18]. Scaffolds were aligned to their host genome using BLAT [19]. Low-quality and chimeric assemblies were filtered out. Alignments of the high-quality scaffolds remaining were compared to the transcriptome annotations in the reference genomes. We counted the total number of unique bases in these alignments, but did so in two different ways, depicted in Fig. 2 as “genome” or “exome”. For the former we counted everything, but for the latter we only counted transcriptome bases within the annotated exons.

Our a priori expectation was that the total number of unique bases would asymptote as the sequencing depth increased. For the exome curves, this was universally observed, typically at 2~8 Gbp, almost regardless of species analyzed. For the genome curves, such asymptotic behavior was best observed in *Drosophila melanogaster*, but not at all in *Homo sapiens*, with other species falling somewhere between these two extremes of behavior. It is not clear when the *Homo sapiens* genome data might asymptote. For *Homo sapiens*, the genome data is nearly seven times the exome data at 16 Gbp of sequencing depth. Even in the curves that reach the asymptote, it is not because the genome or annotated exome has been completely covered, as only 60% of the genome bases and 75% of the exon bases have transcripts aligned against them in the most complete case (*Drosophila*). Given our stringent alignment criteria, we believe the assembled sequences are genuine transcripts. Why so many are not in the “official” annotations is best deferred to the discussions.

**Fig. 3:**
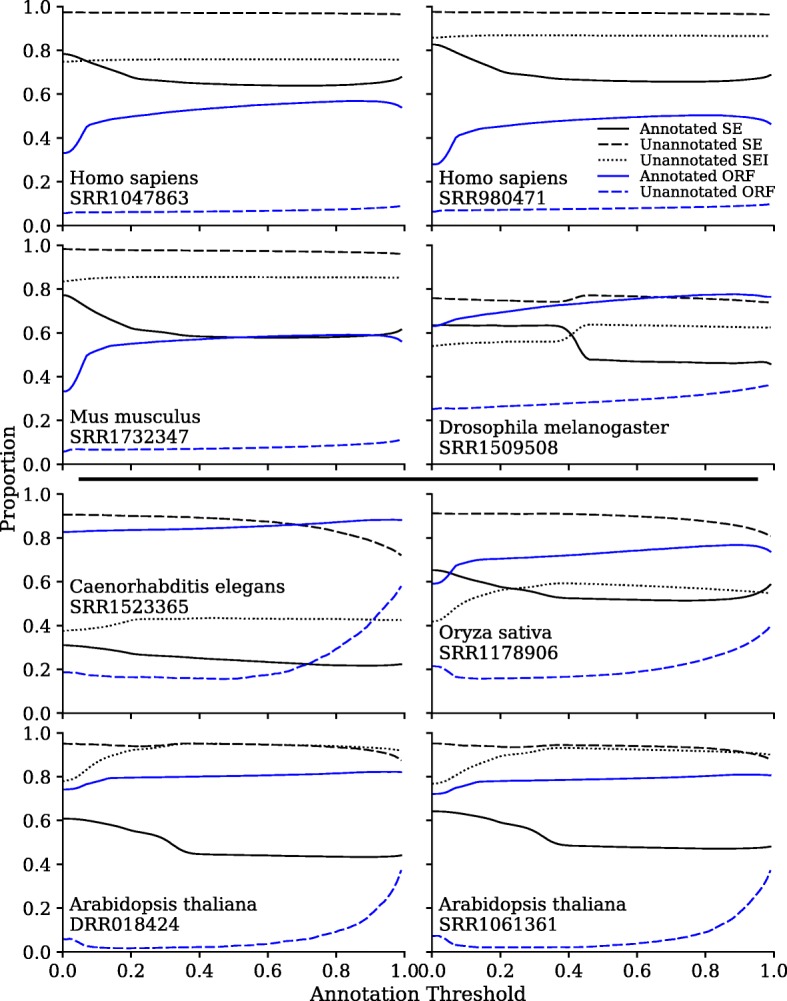
Single-exon and ORF proportions in annotated and unannotated scaffolds. Many scaffolds align partially to the exome. A scaffold is said to be “annotated” when it has an alignment that exceeds an arbitrary fraction, i.e. annotation threshold, of either the scaffold length or of the reference transcript length. Annotated and unannotated SE refers to the proportion of annotated and unannotated transcripts that are single-exon. Unannotated SEI refers to the proportion of unannotated single-exon transcripts that are intronic. Annotated and Unannotated ORF refers to the proportion of scaffolds in each category that have ORFs of at least 100 amino acids in length, out of the scaffolds that are at least 300 bases long

An important consideration is the proportion of the assembled sequences that align with and without introns. This is because the latter case, single-exon transcripts, can arise from a variety of sources including protein-coding genes, long non-coding RNAs (lncRNAs), and improperly spliced pre-mRNAs. The results for assemblies at 16Gbp of sequencing are shown in Fig. 3. For almost all species, unannotated transcripts were overwhelmingly single-exon, essentially 100%, with the exception of *Drosophila melanogaster*. A much smaller proportion of annotated transcripts were classified as single-exon, 50 to 80% depending on parameter settings. In *Homo sapiens*, *Mus musculus*, and *Arabidopsis thaliana* libraries, a majority of the unannotated single-exon material is intronic, suggesting but not proving that they are simply unprocessed mRNAs. We would however be cautious about overly interpreting the single-exon fractions, as incomplete assemblies can produce single-exon transcripts, despite their underlying genes having multiple exons. That said, the difference between the proportions for unannotated and annotated transcripts is striking.

Of further interest is whether the annotated and unannotated scaffolds might contain viable open reading frames (ORFs). In analyzing the scaffolds for ORFs, we only considered scaffolds with a minimum length of 300 bases and looked for ORFs of at least 100 amino acids. As shown in Fig. 3, the annotated scaffolds contain a higher proportion of ORFs than the unannotated scaffolds in all cases. Some species contain higher levels of ORFs in the unannotated scaffolds, which may be partially explained by missing annotations in the references. The proportion of annotated scaffolds not containing ORFs is likely affected by the completeness of the assembly. Transcripts that are partially assembled into separate scaffolds are less likely to have ORFs of sufficient length, even though they would be considered annotated.

The observed differences in genome sequence recovered relative to exome in Fig. 2 are consistent with the total amounts of non-minimal introns [20] in the underlying genomes. *Caenorhabditis elegans* and *Arabidopsis thaliana* are noted for their small genomes (100 Mbp and 120 Mbp respectively) and small proportions of introns greater than a kilobase. *Drosophila melanogaster* has a comparably small genome of 144 Mbp, but larger proportions of introns of kilobase length or larger. *Oryza sativa* has a slightly larger genome of 383 Mbp, but its introns are shorter in length than *Drosophila*. *Homo sapiens* and *Mus musculus* have much larger genomes (3.2 Gbp and 2.8 Gbp respectively) and comparatively large proportions of introns of 10 kilobases in length or larger, with some reaching a megabase in length.

### HiSeq and DNBseq™ platforms are nearly equivalent except in the most GC-rich regions

Here, we compare two short-read NGS sequencing platforms, the market-leading Illumina platform, HiSeq, and a recent platform from MGI, DNBseq™, based on technology from Complete Genomics. Our primary interest is to recover as many complete transcript sequences as possible using a de novo assembly. Within the context of the previous discussion, and in particular Fig. 2, we are most interested in recovering the exome that appears in the genome annotations. Obviously, this will be a function of sequencing depth, but given an equivalent amount of sequencing, might there be significant differences between platforms?

#### Datasets

All of the analyzed sequence was for libraries created from the Universal Human Reference RNA (UHRR), which is comprised of RNA from ten human cell lines, and is commonly used as a control for microarray gene-expression experiments. Nine libraries were constructed by MGI with the MGIEasy RNA Library Prep Set (1000006383, 1000006384) kit, consisting of three sets with three replicates each. Nine sequencing runs with paired-end 100 bp (PE100) data were performed on the BGISEQ-500, giving a total of 416,161,025 reads. Five approximately PE100 runs from the Illumina HiSeq-2000 were used for comparison; they totaled 525,070,317 reads. Notice however that the HiSeq libraries were produced in two different labs. Table 3 shows the datasets used.

**Table 3:** Datasets used to compare DNBseq™ and HiSeq platforms

SRA Run ID	Lab	Platform	# of Spots	# of Bases	Date Published	End Length
ERR1831362	1	BGISEQ-500	48 148 821	9 629 764 200	2017-02-21	100 bp
ERR1831363	1	BGISEQ-500	29 782 959	5 956 591 800	2017-02-21	100 bp
ERR1831364	1	BGISEQ-500	54 940 056	10 988 011 200	2017-02-21	100 bp
ERR1831365	1	BGISEQ-500	36 073 210	7 214 642 000	2017-02-21	100 bp
ERR1831366	1	BGISEQ-500	43 664 065	8 732 813 000	2017-02-21	100 bp
ERR1831367	1	BGISEQ-500	55 025 946	11 005 189 200	2017-02-21	100 bp
ERR1831368	1	BGISEQ-500	53 296 161	10 659 232 200	2017-02-21	100 bp
ERR1831369	1	BGISEQ-500	65 455 754	13 091 150 800	2017-02-21	100 bp
ERR1831370	1	BGISEQ-500	29 774 053	5 954 810 600	2017-02-21	100 bp
SRR1261168	2	HiSeq 2000	134 921 154	26 984 230 800	2014-04-24	100 bp
SRR1261170	2	HiSeq 2000	72 897 482	14 579 496 400	2014-04-24	100 bp
SRR950078	3	HiSeq 2000	100 387 010	20 278 176 020	2013-08-29	101 bp
SRR950080	3	HiSeq 2000	91 781 477	18 539 858 354	2013-08-29	101 bp
SRR950084	3	HiSeq 2000	125 083 194	25 266 805 188	2013-08-28	101 bp

All datasets were sourced from the NCBI and ENA Sequence Read Archives

#### Quality control

SOAPnuke [21] was used to filter the reads based on the amount of low-quality bases, ambiguous bases (Ns), or adapter sequence. In total, the DNBseq™ libraries had 4.70% of their reads filtered (0.25% adapter, 2.83% low-quality, and 1.62% ambiguous); the HiSeq libraries had 27.91% of their reads filtered (1.48% adapter, 24.38% low-quality, and 2.06% ambiguous). Notice however that this large difference in number of low-quality reads filtered can potentially be explained by differences in base callers. It does not necessarily reflect any intrinsic quality difference between the platforms. Detailed results are given in Table 4.

**Table 4:** Read quality filtering on two sequencing platforms

Platform	Name	Adapter		Low Quality		N%	
DNBseq™	ERR1831362	125 926	0.26%	1 424 492	2.96%	769 503	1.60%
	ERR1831363	79 533	0.27%	871 008	2.92%	479 635	1.61%
	ERR1831364	127 069	0.23%	1 476 496	2.69%	869 764	1.58%
	ERR1831365	103 994	0.29%	1 051 568	2.92%	588 427	1.63%
	ERR1831366	110 324	0.25%	1 060 462	2.43%	711 236	1.63%
	ERR1831367	132 461	0.24%	1 581 470	2.87%	901 123	1.64%
	ERR1831368	133 444	0.25%	1 588 843	2.98%	875 865	1.64%
	ERR1831369	152 027	0.23%	1 825 708	2.79%	1 049 309	1.60%
	ERR1831370	83 674	0.28%	898 776	3.02%	484 541	1.63%
HiSeq	SRR1261168	3 497	0.00%	30 319 019	22.47%	466 900	0.35%
	SRR1261170	509	0.00%	33 381 937	45.79%	6 147 822	8.43%
	SRR950078	1 313 463	1.31%	20 004 668	19.93%	1 307 643	1.30%
	SRR950080	2 022 559	2.20%	18 689 016	20.36%	1 194 269	1.30%
	SRR950084	4 443 842	3.55%	25 596 160	20.46%	1 675 649	1.34%

The columns show the number and percentage of reads filtered out based on them containing adapter sequence, having too many low quality bases, and having too many ambiguous bases

To reduce the effect of differences in library preparations, we also filtered the reads by aligning against the Genome Reference Consortium human genome build38 (GRCh38) with HISAT2 [22]. This removed likely contaminations, and eliminated the spike-in reads that were added to some libraries. For DNBseq™ libraries, 3.97% of remaining reads could not be aligned; for HiSeq libraries, 3.68% of remaining reads could not be aligned. To reduce differences resulting from input material amounts or PCR cycles, we also filtered duplicate reads using Picard Tools [23]. For DNBseq™ libraries, 27.23% of remaining reads were filtered out; for HiSeq libraries, 28.85% of remaining reads were filtered out. Full results are given in Table 5.

**Table 5:** Read filtering for differences in library preparation

		Preprocessing			
Platform	Name	Raw	Filtered	Mapped	Deduplicated
DNBseq™	ERR1831362	48 148 821	45 828 900	44 015 469	32 497 907
	ERR1831363	29 782 959	28 352 783	27 237 354	21 102 998
	ERR1831364	54 940 056	52 466 727	50 421 993	36 693 200
	ERR1831365	36 073 210	34 329 221	32 932 438	24 745 818
	ERR1831366	43 664 065	41 782 043	40 108 615	29 250 048
	ERR1831367	55 025 946	52 410 892	50 302 197	36 153 502
	ERR1831368	53 296 161	50 698 009	48 688 107	34 475 744
	ERR1831369	65 455 754	62 428 710	59 984 288	41 622 545
	ERR1831370	29 774 053	28 307 062	27 164 676	20 606 457
HiSeq	SRR1261168	134 921 154	104 132 308	101 903 998	70 430 950
	SRR1261170	72 897 482	33 367 214	32 597 422	27 498 074
	SRR950078	100 387 010	77 761 236	73 979 293	50 505 406
	SRR950080	91 781 477	69 875 633	66 896 955	49 310 706
	SRR950084	125 083 194	93 367 543	89 192 069	61 653 799
DNBseq™	Total	416 161 025	396 604 347	380 855 137	277 148 219
	% Removed		4.70%	3.97%	27.23%
HiSeq	Total	525 070 317	378 503 934	364 569 737	259 398 935
	% Removed		27.91%	3.68%	28.85%

Here we show reads remaining after each preprocessing step. The columns indicate read counts after SOAPnuke filtering (Filtered), aligning to GRCh38 with HISAT2 (Mapped), and PCR deduplication with Picard Tools (Deduplicated)

#### Assembly results

**Fig. 4:**
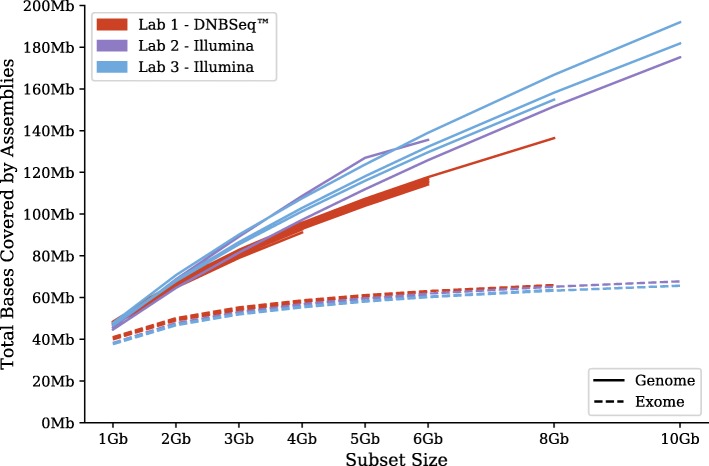
Genome vs exome coverage from HiSeq and DNBseq™. Each line corresponds a subsampled library, with total sizes of 1, 2, 3, 4, 5, 6, 8, and 10 Gbp

We performed multiple de novo assemblies, all using SOAPdenovo-Trans [18], with randomly-selected subsets of each library. Target sizes were 1, 2, 3, 4, 5, 6, 8, and 10 Gbp, to the extent that sufficient data was available in the source library. These assemblies were aligned against GRCh38 with BLAT [19] and evaluated against the GENCODE v28 [24] annotations. As before we differentiate between alignments to the genome and exome. Results are shown in Fig. 4. Looking only at the genome curves, the HiSeq libraries appear superior; but looking at the exome curves, there is no appreciable difference between platforms. Each user will have to decide for him/herself if this additional genome coverage is worthwhile, given that it was not included in the exome annotations from GENCODE.

Next, the scaffolds were aligned against the GRCh38 transcriptome using the LAST aligner [25], to evaluate the completeness of the RNA-seq assembly. To be declared complete, at least 95% of the annotated transcript must be aligned to by a single RNA-seq scaffold. This definition recognizes that a complete RNA-seq assembly is often longer than the corresponding annotation, because the former will include UTR sequences while the latter typically does not. The results are depicted in Table 6. DNBseq™ results were fairly consistent in the number of complete transcripts recovered for each subset size. HiSeq results showed greater variation with subset size, which we believe is due to differences in library preparation among different labs. In particular, the libraries SRR1261168 and SRR1261170 seemed of especially high quality, as determined by transcript completeness, and both were generated at the same sequencing center.

**Table 6:** Completeness of transcripts assemblies per library

		Complete Transcripts Assembled									
Platform	Name	1Gbp	2Gbp	3Gbp	4Gbp	5Gbp	6Gbp	8Gbp	10Gbp	12Gbp	20Gbp
DNBseq™	COMBINED	2 483	3 891	4 720	5 276	5 705	5 994	6 458	6 716	6 979	7 472
	ERR1831362	2 049	3 306	4 140	4 719	5 189	5 530				
	ERR1831363	1 981	3 235	4 026	4 641						
	ERR1831364	2 052	3 393	4 253	4 805	5 187	5 527				
	ERR1831365	1 997	3 295	4 125	4 667						
	ERR1831366	1 995	3 293	4 111	4 739	5 193					
	ERR1831367	2 061	3 351	4 173	4 775	5 216	5 511				
	ERR1831368	1 951	3 304	4 131	4 707	5 164	5 471				
	ERR1831369	2 032	3 323	4 170	4 772	5 178	5 575	5 989			
	ERR1831370	1 920	3 260	4 091	4 622						
HiSeq	SRR1261168	2 363	3 696	4 483	4 987	5 439	5 720	6 304	6 688	6 933	
	SRR1261170	1 908	3 135	3 908	4 478	4 916	5 107				
	SRR950078	809	1 402	1 820	2 190	2 444	2 673	3 024	3 362		
	SRR950080	982	1 620	2 126	2 504	2 816	3 094	3 471			
	SRR950084	936	1 573	2 068	2 433	2 722	2 985	3 379	3 666		

Complete transcript counts are shown for each randomly-selected subset for each library

We also combined all of the DNBseq™ libraries and assembled subsets of different sizes. The results showed that the combined library performs better in terms of complete transcripts than any other DNBseq™ library at every subset size (see COMBINED row in Table 6). This is likely because the sequences that are sampled in different libraries are complementary and occur in sufficient quantity such that they will be assembled in the combined libraries.

**Fig. 5:**
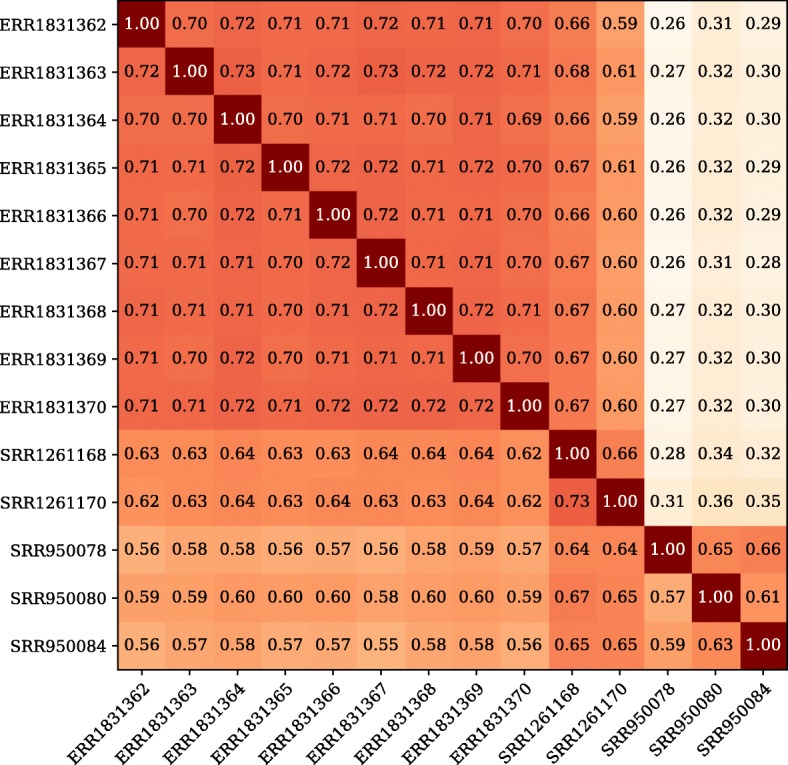
Overlap in complete assembled transcripts. Comparisons of complete transcripts between libraries at the 4 Gbp subsets. For the libraries in each row, the fraction is calculated as the number of complete transcripts that are shared with the library from each column, divided by the total number of complete transcripts for the library in that row

To get an idea of the overlap in complete assembled transcripts between the two sequencing platforms, we compared the complete transcripts for the 4 Gbp subset assemblies, as that was the largest available subset in most of the libraries. Results are depicted in Fig. 5. Complete overlap does not exist. This was the case whether comparing libraries from different sequencing platforms or from the same sequencing platform. The implication is that we are mostly seeing the inevitable differences in sampling of lower level transcripts. However, the amount of overlap between libraries from the same platform was higher than that between libraries from different platforms, indicating that some transcripts are more likely to be completely assembled when we use a particular sequencing platform.

**Fig. 6:**
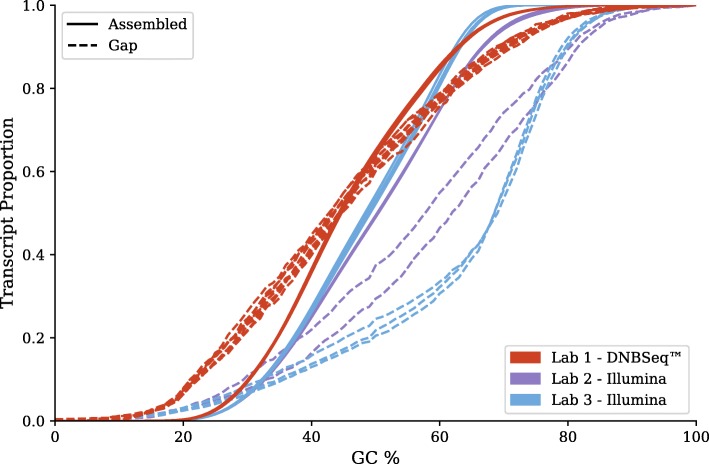
GC-content of assembled versus gap regions. Cumulants of GC-content in the assembled versus gap regions for (a) DNBseq™ libraries and (b) HiSeq libraries. Each pair (solid and dotted lines) represents a single library

Next, we looked at the 4 Gbp assemblies for transcripts that were not complete in any library on one sequencing platform, but complete for at least one library on the other platform. We then compared the GC contents of the assembled regions to the gap regions. The results are shown in Fig. 6. For DNBseq™ libraries, gap regions are uniformly distributed among low, mid, and high GC-content. In contrast, albeit for only some of the HiSeq libraries, the gap regions reveal a bias against GC-rich sequence. The fact Illumina libraries can be susceptible to both high and low GC biases has previously been reported [10–12], although there are techniques that can reduce the magnitude of the biases. And indeed, the two best HiSeq libraries from our study showed much less of a GC-content bias than the other three.

**Fig. 7:**
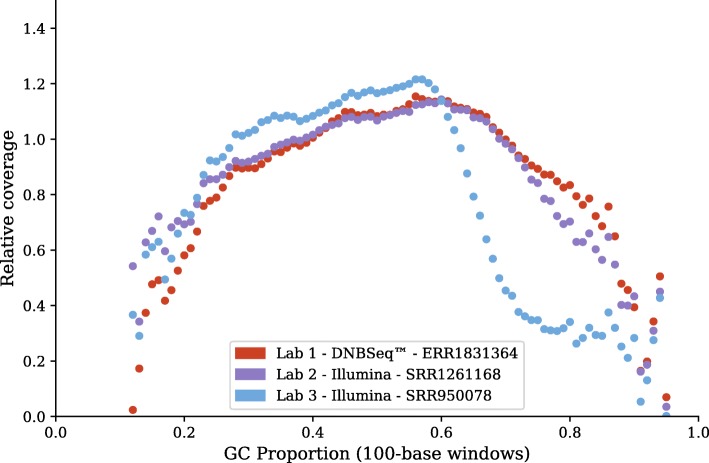
GC-bias plots for representative libraries. Relative coverage as a function of GC-content, computed on 100-base windows across the set of 565 transcripts described in the text. A relative coverage of one would indicate no bias

Looking further into the GC-content biases, we examined the read depths from the HISAT2 alignments. We established a set of 565 non-overlapping transcripts with a minimum average depth of 10 across all our 4 Gbp datasets. To ensure that the subset of transcripts are representative of the complete set of transcripts, we counted the number of transcripts with GC-content in 1% segments for both sets. These two sets have a Spearman correlation of 0.904, showing that the subset has a similar GC-content distribution to the full set of transcripts. Then, for each 100 base pair window along each transcript, we determined their GC-content and average read depth. The ratio of each window depth to the entire transcript average depth is taken. Figure 7 depicts the average of these ratios for each GC-content bin from 0 to 100% on a few representative libraries. The DNBseq™ library ERR1831362 and the HiSeq library SRR1261168 look rather similar, not surprisingly considering that these were among the best in terms of assembly completeness. However the HiSeq library SRR950078 exhibited a substantial drop-off in read depth at high GC-content, consistent with its inferior assembly completeness.

**Fig. 8:**
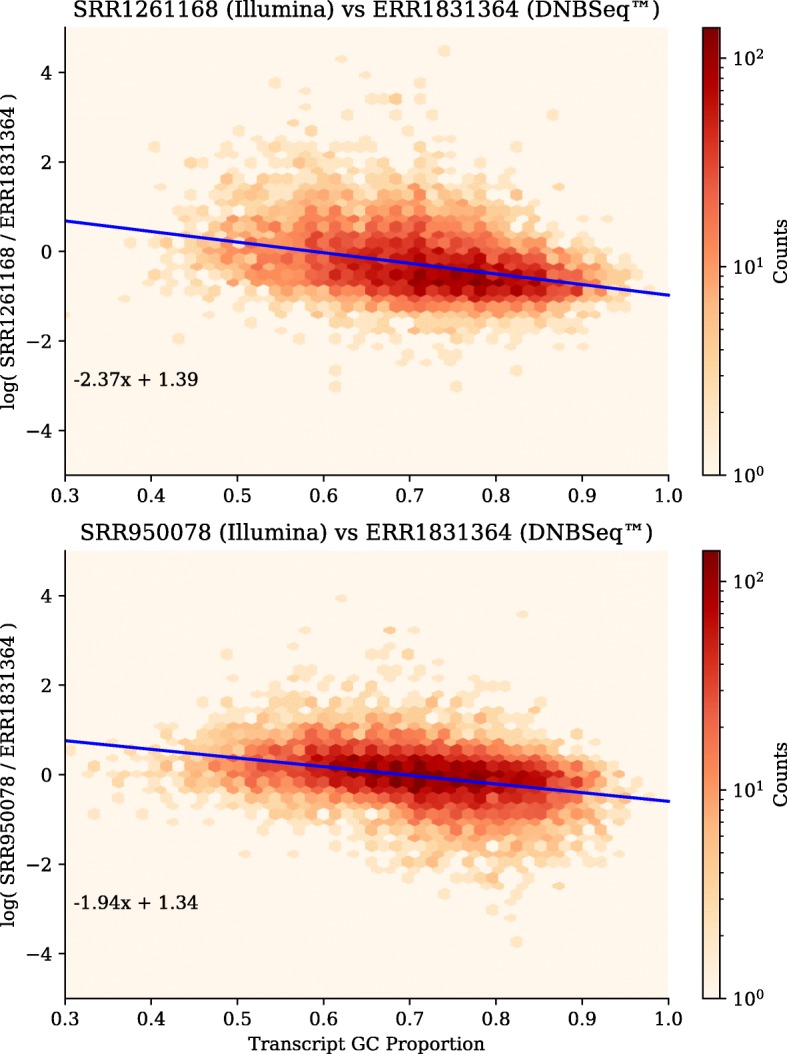
A slight bias in the transcript abundances vs GC-content. The log ratio of the expression levels of (a) SRR1261168, the HiSeq run with the most complete assemblies and (b) SRR950078, the HiSeq run with the least complete assemblies, compared to ERR1831364, the DNBseq™ run with most the complete assemblies. Regression fit is shown in blue, with numerical parameters indicated

Finally, we estimated the transcript abundances using Kallisto [26], focusing again on the 3 representative libraries, plotting the ratio of transcript abundances from the DNBseq™ library to each of the two HiSeq libraries. These results are shown in Fig. 8. Transcripts were only included when their estimated abundances were at least 10 transcripts per million in both libraries, to avoid the higher-variance low-abundance transcripts. Comparing the 9,687 transcripts (4.75% of all transcripts) with abundances meeting the threshold in ERR1831367 and SRR1261168, we see a slight slope in the regression-fitted line, as expected if there is a bias against higher GC-content reads in the HiSeq data. Comparing the 11,753 transcripts (5.77% of all transcripts) with abundances meeting the threshold in ERR1831367 and SRR950078, we see a similar slope, consistent with the other comparison.

## Discussion

*How much RNA-Seq data is optimal?* It is well-known that there are diminishing returns to ever deeper transcriptome sequencing and the exact choice will always be a function of budget vs ambition. However, it is less well-known that deeper transcriptome sequencing will generate a plethora of single-exon transcripts that are not typically included in most genome annotations, especially but not exclusively for human sequencing. What these additional transcripts might be has long been a subject of debate. The first high-profile mention of this phenomenon was the FANTOM Consortium publication of full-length mouse cDNAs, where it was claimed that 15,815 of 33,409 non-redundant cDNAs were non-protein-coding RNA genes [27]. However, most of these genes were poorly conserved across mammalian evolution [28] and it was unclear how many were biologically functional. These debates escalated when the ENCODE Consortium assigned biochemical functions to 80% of the human genome [29]. The arguments focussed on the definition of biological functionality, and the relevance or not of evolutionary conservation [30–34]. We do not wish to revisit these arguments. The point of Figs. 2 and 3 was simply to demonstrate what you get if you sequence more deeply on an RNA-Seq library. It is up to the individual user to decide if such additional transcripts are worth the extra expenditures.

*Which sequencing platform is better?* For most users, next-generation sequencing is a choice between higher-quality shorter reads, as exemplified by the market-leading Illumina platform, or lower-quality longer reads, as exemplified by Pacific Biosciences and Oxford Nanopore. In the former category, the most pertinent question is if the DNBseq™ platform (BGISEQ-500 and more recent MGISEQ-2000 and MGISEQ-T7, which are capable of PE150 reads) is a viable alternative to Illumina. Here, we show that for recovery of transcript sequences from de novo assembled RNA-Seq libraries the two platforms give equally good results. Some of the Illumina libraries under-represented GC-rich sequences, leading to gaps in the assemblies. However, other Illumina libraries did not exhibit such a GC-content bias, and without a systematic analysis of library making protocols that is beyond the scope of this publication, it is unclear if this is an intrinsic disadvantage of the Illumina platform. However, the importance of good library construction, and the consequent biases if this is not done right, is worth emphasizing regardless of sequencing platform used.

## Conclusions

Increasing sequencing depth of RNA-Seq experiments has quickly diminishing returns in terms of exomic sequence assembled. A large portion of the additional sequences assembled as sequencing depth increases appears to be unannotated single-exon transcripts. Of these sequences, a majority appears from intronic regions.

DNBseq™ is a viable alternative to HiSeq for de novo RNA-Seq assembly. Libraries sequenced on both technologies recovered similar numbers of full-length transcripts after assembly. Higher levels of GC-bias are seen in some of the Illumina libraries, which is likely attributable to differences in library preparation.

## Methods

### Increased sequencing depth enriches for single-exon transcripts not reported in genome annotations

#### Quality control

Reads are trimmed by Trim Galore! 0.3, which removes adapter/primer sequences. The --paired parameter is used, with the --quality parameter set to 0 so that no bases are removed for low-quality scores (these are dealt with later). To improve alignment rates in the next step, five bases were also trimmed from the 5′-ends of reads in the SRR1523365 (*C. elegans*) data.

Each library is split into million read-pair chunks. Each chunk is aligned to their reference genome by Tophat 2.0.13, using the --b2-very-sensitive parameter to increase alignment sensitivity. Reads are considered “clean” if both ends align and they are consistent in location and direction. Clean reads from all chunks are merged and used for further processing.

#### Assembly and alignment

Clean read-pairs from each library are randomly subsampled to generate 1, 2, 3, 4, 5, 6, 8, 10, 12, 14, and 16 Gbp of sequencing data. These subsets are assembled with SOAPdenovo-Trans 1.03 (2014-01-23) using default parameters, but with the -F argument set to enable gap filling and the average insert size defined as 250 bp. Post-processing with GapCloser filled in more gaps.

Assembled sequences are aligned against their host genome by BLAT [19] using the -fine (looks harder for smaller initial and terminal exons) and -ooc (speeds up alignment by skipping overly common 11-mers) parameters. Scaffolds that align to the genome over at least 98% of their length are deemed to be correctly assembled. Alignments are compared with the reference annotations for the host genome.

To compute the proportion of unannotated and annotated scaffolds with single vs multi-exon alignments, we set a percentage threshold that the alignment must reach, from 0 to 100% at 1% intervals. A scaffold is considered to be annotated if has an alignment that is greater than that percentage threshold, where the denominator on the percentage calculation is the length of the scaffold or the length of the reference transcript (whichever is more favorable).

To analyze the proportion of unannotated and annotated scaffolds containing viable ORFs, we looked at the set of scaffolds at least 300 bases long in each category, and computed the proportion of those that had ORFs at least 100 amino acids long.

### HiSeq and DNBseq™ platforms are nearly equivalent except in the most GC-rich regions

#### Quality control

Reads from each library are first filtered with SOAPnuke 1.5.6. We chose filtering parameters matching the previous study of a reference human genome dataset generated on DNBseq™ [35]. Reads are eliminated when more than 10% of the bases have a PHRED score of less than 10 or when more than 1% of the bases are ambiguous N’s. Reads containing at least 50% of an adapter with no more than one mismatch are also filtered.

To reduce the effect of contamination or otherwise unwanted sequence, we filter the reads by aligning them to the Genome Reference Consortium human genome build 38 (GRCh38) using HISAT2 2.1.0 and default parameters. Both ends must be aligned concordantly for the read to be kept. This removes contamination, spike-in reads, etc.

PCR duplicates are marked by the Picard Tools 2.18.5 MarkDuplicates command, and then filtered using Samtools 1.8 and an awk command to reduce the effect of differences in input materials and PCR cycles between libraries.

#### Assembly and alignment

The clean deduplicated reads are randomly subsampled from each library to generate 1, 2, 3, 4, 5, 6, 8, and 10 Gbp of sequencing data, to whatever extent that the desired amount of data is available. The datasets are assembled with SOAPdenovo-Trans 1.04 using default parameters, with the -F argument set to enable gap filling and the average insert size set to 200 bp. This is followed with GapCloser post-processing to fill in more gaps. Assembled sequences are aligned against the GRCh38 genome with BLAT 36 [19], using parameters -fine (looks harder for smaller initial and terminal exons) and -ooc = 11.ooc (speeds up alignment by skipping overly common 11-mers). Scaffolds aligning to the genome above 98% of their length are said to be assembled correctly. Their alignments are compared with the Gencode v28 [24] annotations to determine exome vs genome coverage.

#### Completeness

Assembled sequences are also aligned with lastal 941 [25], using default parameters, to the set of transcripts in GRCh38 generated from the Gencode v28 annotations. For a reference transcript to be considered complete, there must be a scaffold that can align over 95% of that reference transcript. Only scaffolds which have an unambiguous top-scoring alignment are considered.

#### GC content gaps/biases

GC content analysis is done on the 4 Gbp subsets, because all libraries except for SRR515084 have a 4 Gbp subset. For comparing assembled versus gap regions, transcripts are chosen if they are not assembled in any other library from the same sequencing platform, but assembled in a library from the other sequencing platform. Regions are declared to be a gap when there are no scaffolds that align to that region of the annotated transcript and, moreover, there are no reads (assembled or unassembled) that align to that region. Only transcripts with at least 10 bases worth of gap region are used for our comparisons.

To analyze GC-bias in reads coverage, we must exclude genes with multiple splice variants, because their reads coverage cannot be confidently assigned. Hence we limit the analysis to a set of 565 transcripts that have no overlap with any other annotated transcripts and that also have a minimum average read depth of 10 across their lengths. For each 100 bp window along each transcript, we calculate the ratio of the read depth for that window against the average read depth along the transcript, as well as the GC-content of that window. The set of all depth ratios is averaged and plotted against GC-content.

To analyze the effect of GC-bias on transcript abundance, we run Kallisto 0.44.0 [26] to estimate transcript abundance. For each library pair, we plot the ratio of the transcript abundance for each transcript, as a function of GC-content. Only transcripts that have a transcripts per million (TPM) estimate of 10 or greater are included.

## Data Availability

All sequencing data is used in this study was previously available on SRA, with the identifiers described in the datasets sections. Supporting code is available as follows. Project name: Supporting code for “Impact of sequencing depth and technology on de novo RNA-Seq assembly”. Project home page: https://github.com/gwonglab/rnaseq_depth_and_technology/releases/tag/Paper [36]. Operating system(s): Linux. Programming Language: Python, Snakemake. Requirements: Conda, Snakemake, Trim Galore!, Tophat2, SOAPdenovo-Trans, GapCloser, BLAT, Python. License: GPL 3.0.

## Abbreviations

bp: base-pair
cDNA: complementary DNA
cPAS: combinatorial Probe Anchor Synthesis
DNB: DNA nanoballs
Gbp: Gigabase-pairs
GRCh38: Genome reference consortium human genome build 38
NCBI: National Center for Biotechnology Information
NGS: Next generation sequencing
nm: nanometer
PCR: Polymerase chain reaction
PE100: Paired-end 100 bp
RNA-Seq: RNA sequencing
SRA: Sequence Read Archive
UHRR: Universal human reference RNA
UTR: Untranslated region
